# Supplementary material for: Antenatal screenings and maternal diagnosis among pregnant women in Sao Tome & Principe—Missed opportunities to improve neonatal health: A hospital-based study
Source: PLOS Glob Public Health. 2022 Dec 29;2(12):e0001444. doi: 10.1371/journal.pgph.0001444 (PMC10021443; doi:10.1371/journal.pgph.0001444)
Supplement: Attachment — Submitted filename: Response to Reviewers PLOS_ANC_22-00754.docx [file pgph.0001444.s002.docx]

Rebuttal letter

Point-by-point response to Reviewers

Dear Editor and Reviewers,

On behalf of all authors, we express our gratitude to you for the constructive review of our manuscript (PGPH-D-22-00754) entitled “Antenatal screening tests and antenatal problems among pregnant women in Sao Tome and Principe: a hospital-based study”.

We have revised the manuscript based on all the comments raised by the Editor and Reviewers 1 and 2.

In the following detailed response, we address each comment calling for changes point-by-point, indicating where relevant additional texts have been added to the body of the manuscript.

We submit a manuscript with track changes and a clean version of the review manuscript.

We believe that with the changes made, the manuscript has improved substantively in view of being considered for publication.

___________________________________________________

**Journal Requirements:**

1. Please include a complete copy of PLOS’ questionnaire on inclusivity in global research in your revised manuscript. Our policy for research in this area aims to improve transparency in the reporting of research performed outside of researchers’ own country or community. The policy applies to researchers who have travelled to a different country to conduct research, research with Indigenous populations or their lands, and research on cultural artefacts. The questionnaire can also be requested at the journal’s discretion for any other submissions, even if these conditions are not met.  Please find more information on the policy and a link to download a blank copy of the questionnaire here: https://journals.plos.org/globalpublichealth/s/best-practices-in-research-reporting. Please upload a completed version of your questionnaire as Supporting Information when you resubmit your manuscript

Authors’ response:

PLOS’ questionnaire on inclusivity in global research is now submitted.

2. In the online submission form, you indicated that "The datasets used and/or analyzed during the current study are available from the corresponding author on reasonable request.". All PLOS journals now require all data underlying the findings described in their manuscript to be freely available to other researchers, either 1. In a public repository, 2. Within the manuscript itself, or 3. Uploaded as supplementary information.

This policy applies to all data except where public deposition would breach compliance with the protocol approved by your research ethics board. If your data cannot be made publicly available for ethical or legal reasons (e.g., public availability would compromise patient privacy), please explain your reasons by return email and your exemption request will be escalated to the editor for approval. Your exemption request will be handled independently and will not hold up the peer review process but will need to be resolved should your manuscript be accepted for publication. One of the Editorial team will then be in touch if there are any issues.

Authors’ response:

The datasets used and analyzed during the current study are all available within the manuscript itself.

3. Please upload all main figures as separate Figure files in .tif or .eps format only and remove the embedded figures from the manuscript file. For more information about how to convert and format your figure files please see our guidelines:

<https://journals.plos.org/globalpublichealth/s/figures>

Authors’ response:

Figure 1 (revised) is now submitted separately as TIF format.

4. We do not publish any copyright or trademark symbols that usually accompany proprietary names, eg (R), (C), or TM (e.g. next to drug or reagent names). Please remove all instances of trademark/copyright symbols throughout the text, including © on pages 12 and 15.

Authors’ response:

Done.

Additional Editor Comments (if provided):

Thanks for submitting your manuscript for consideration. A major revision is required prior to a final decision.

Authors’ response:

We truly appreciate the Editor and Reviewer´s feedback and contributions.

We now submit a revised manuscript. We believe that with the major changes made the manuscript has improved substantively and will hope that now it will be accepted for publication.

**Reviewers' comments:**

Reviewer's Responses to Questions

**Comments to the Author**

1. Does this manuscript meet PLOS Global Public Health’s [publication criteria](https://journals.plos.org/globalpublichealth/s/criteria-for-publication)? Is the manuscript technically sound, and do the data support the conclusions? The manuscript must describe methodologically and ethically rigorous research with conclusions that are appropriately drawn based on the data presented.

Reviewer #1: Partly

Reviewer #2: Partly

Authors’ response:

We truly appreciate the Reviewer´s feedback and contributions.

We now submit a revised manuscript and added important information to the Materials and Methods section.

2. Has the statistical analysis been performed appropriately and rigorously?

Reviewer #1: Yes

Reviewer #2: No

Authors’ response:

We now submit a statistical analysis and new tables according to the reviewers’ suggestions. We believe that with the changes made the manuscript it is now improved substantively.

3. Have the authors made all data underlying the findings in their manuscript fully available (please refer to the Data Availability Statement at the start of the manuscript PDF file)?

The [PLOS Data policy](https://journals.plos.org/globalpublichealth/s/data-availability) requires authors to make all data underlying the findings described in their manuscript fully available without restriction, with rare exception. The data should be provided as part of the manuscript or its supporting information, or deposited to a public repository. For example, in addition to summary statistics, the data points behind means, medians and variance measures should be available. If there are restrictions on publicly sharing data—e.g. participant privacy or use of data from a third party—those must be specified.

Reviewer #1: Yes

Reviewer #2: Yes

Authors’ response:

Correct. The datasets used and analyzed during the current study are all available within the manuscript itself.

4. Is the manuscript presented in an intelligible fashion and written in standard English?

PLOS Global Public Health does not copyedit accepted manuscripts, so the language in submitted articles must be clear, correct, and unambiguous. Any typographical or grammatical errors should be corrected at revision, so please note any specific errors here.

Reviewer #1: Yes

Reviewer #2: Yes

Authors’ response:

An additional effort was made with the help of an English-speaking health professional who assisted in improving the language of the paper now submitted.

**5. Review Comments to the Author**

Please use the space provided to explain your answers to the questions above. You may also include additional comments for the author, including concerns about dual publication, research ethics, or publication ethics. (Please upload your review as an attachment if it exceeds 20,000 characters)

**Reviewer #1:**

The manuscript, entitled “Antenatal screening tests and antenatal problems among pregnant women in Sao Tome and Principe: a hospital-based study”, used a retrospective study design to review the ANC cards of 518 pregnant women. The purpose of the study was to identify screenings performed and antenatal problems with a focus on how to improve the ANC in STP to reach the newborn health post-2015 Sustainable Development Goals. The study found that the ANC screening service was less than optimal. Specifically, pregnant women had no screening done for HIV in 7.6%, syphilis in 10%, HBV 40.2%, malaria 27%, haemoglobin 37.5%, blood glucose 46.2%, urine test 31%, stool exams 29% and 42.1% had no ultrasound.

In my opinion, the study is worthwhile considering recent calls to focus on quality of care and not just coverage of essential indicators. The study shows the coverage-quality gap in STP indicators for maternal health services and confirms what has already been seen around the world about the coverage-quality gap

The manuscript is well written in standard English, and the methodology used in the study is sound, and the details are well elaborated. Nevertheless, there are some minor concerns that need to be addressed to make the manuscript scientifically suitable for publication in PLOS Global Public Health. The concerns are outlined below according to the sections:

**General comments**
Authors should ensure that the manuscript is proofread and issues of grammar and wrong spelling are rectified. In addition, there should be consistency in the English used, either American or British, but not both, which is obvious in the manuscript.

Authors’ response:

We now submit a revised manuscript with American style. Issues of grammar and wrong spelling were all rectified.

In this revised paper we submit a new title as we believe that it better describes and addresses the global picture of this study:

Title: “Antenatal screenings and maternal diagnosis among pregnant women in Sao Tome & and Principe - missed opportunities to improve neonatal health: a hospital-based study”

**Introduction**

Line 58-60. There is a problem with the sentence structure. The phrase “should be provided” has to be deleted for the sentence to flow well.

Authors’ response:

Done.

Line 68- 70. Correct the pronoun “it” in the sentence to reflect the use of the pleural form of “infections” [in pregnancy]. A similar issue appears in line 78-79 where “go” is used for the singular form of “sickle cell disease”

Authors’ response:

Done.

Line 89-91. I don’t think it is true that STP lacks skilled medical personnel and health infrastructure. They may be present but woefully inadequate. Indeed, lack and inadequate are not the same, and it is unlikely that it is the case of the forma in STP. Clearly, the current study was health facility based, and that alone is enough to disqualify the use of “lack”. Furthermore, a hospital is regarded as a health infrastructure, and in this manuscript, it is indicated that the majority of births take place in the only hospital in STP.

Authors’ response:

The sentence was deleted.

Line 100. There is an issue with grammar. “Studies” is pleural. Therefore, check the use of “analyses”. It is better to use most studies… analyze or analyse this service…, instead of analyses.

Authors’ response:

Done.

Line 103. The WHO document on positive pregnancy experience is referred to in the manuscript. Authors should, therefore, comply and use contacts and not visits as recommended. That should be corrected throughout the manuscript

Authors’ response:

Done. The term “contacts” is now used throughout the manuscript.

**Materials and methods**

Line 114-115. Correct sentence structure. Authors should also acknowledge that the study was purely descriptive in nature as part of the study design.

Authors’ response:

Done.

Line 171-172. Refer to my earlier comment on the use of ANC visits.

Authors’ response:

Done.

It is conceptually appropriate to place the data source section before the variable section.

Authors’ response:

Done.

Nowhere in the methods section has it been mentioned what was used to extract the information for the ANC record cards.

Authors’ response:

The information was now added.

Then a questionnaire is mentioned towards the end under data management. What was this questionnaire used for?

Authors’ response:

The sentence was deleted. Data management and quality section was updated.

Line 217. What does it mean to put percentages in parenthesis after the mention of frequencies? The two are obviously not the same and do not provide the same information.

Authors’ response:

The sentence was corrected.

Authors indicated that anonymity and the safety of participants were ensured. How was safety ensured in the study?

Authors’ response:

Safety was not an issue in this study. This information was deleted.

**Results**
Line 239-241. The sentence is confusing? Is the mean age and standard deviation for the maternal age or the age of the pregnancy? The range shows a maximum of 43 years. I don’t think a pregnancy can last that long. The following information shows the information is about age, so I wonder why “current pregnancy” was mentioned in the beginning sentence.

Authors’ response:

The information now provided was amended according to pregnant women´s age.

The participants´ characterization is now presented for a total of 511 to be in accordance with the total number of pregnant women in Tables 3 (n=76), 4 (n=237) and 5 (n=198).

In the first version of this manuscript, a total of 518 participants had their ANC pregnancy card reviewed, however, for the Tables 3, 4 and 5, we had to exclude 7 pregnant women since information on the number of ANC contacts was missing. Therefore, for clarity, in this new revised manuscript we excluded them and corrected the total percentages according to a total of 511 pregnant women. This information was also included in the Materials and Methods.

The variable employment was described in the methods section as employed and not employed. In the results, there is another category—students. Does it mean that students are not employed? If so, why are they not added to the unemployed category or the employed if the respondent is a student and is involved in an income-generating activity?

Authors’ response:

Done. Students were included in the unemployed category.

Here again, visits have been used instead of **contacts.**

Authors’ response:

Done. The term “contacts” is now used throughout the manuscript.

Figure 1 does not provide the information described in the narrative. I can see that the maximum value (percentage) from the figure is 10.0%. How did the authors come up with the 51.7% for the first trimester?

Authors’ response:

We now included a new Figure 1 that addresses the Reviewer 1 suggestions.

The maximum value is 10%. The sum of all percentages from the 4^th^ week until the 13^th^ gestational week is equal to 51.7%.

What was the description used for the trimesters? It is only stated in the methods that information on the timing of the first contact was collected.

Authors’ response:

We have now added information regarding the trimester definition: “For ANC characterization, the following variables were included: timing at first ANC booking on the first (conception to 13th gestational age week), second (14-26 week) or third trimester (27-40 week),…”

The figure should either be compressed into the trimesters or the narrative should capture the weeks. Data labels could also enhance readability.

Authors’ response:

We have now added a new figure differentiating the first, second and third trimesters.

Tables 3, 4, and 5 are confusing. The titles mention “adequacy of use”. Is there any standards for the coverage? What are the criteria for measuring adequacy in this case? Appropriate titles should be given to disease tables to reflect the information they provide.

Authors’ response:

We merged the Tables 3, 4 and 5 into a Table 1 (revised manuscript) for clearer comparison between data, including p-values from the Chi-square test.

Also, are the data being compared in these tables? For example, are the authors comparing the coverage of HIV testing in the 1st test group with the 2nd test group? If comparisons are being made, which appears to be the case, the statistical test needs to be mentioned.

Authors’ response:

Material and Methods section and Results were updated. The revised manuscript includes p-values for the comparison of screenings and different ANC groups.

**Discussion**
Since 2016, the WHO has recommended at least 8 ANC contacts. In the second paragraph of the discussion, the authors said that the minimum number of contacts was four. Is it the case in STP that the new recommendations have not been adopted?

Authors’ response:

We have now included new information. In the Introduction the following sentence was added: “In STP, the transition from the model of four ANC visits to the latest WHO recommendation of eight contacts occurred between 2017 and 2018.”

Therefore, since this study occurred in the period of transition, we presented in the discussion the rates for a minimum of four and a minimum of eight contacts and compared those rates to similar African countries.

Line 336-337. I spot a grammar issue in the sentence that needs to be corrected

Authors’ response:

Done.

Line 339. What does reference no. 8 serve in this situation?

Authors’ response:

The reference was deleted.

Line 344-345. In this context, authors cannot use both either and neither. Please revise the sentence.

Either is used with “or” and neither with “nor”.

Authors’ response:

The sentence was amended according to the Reviewer´s correction.

Line 410. Is “testes” the word?

Authors’ response:

We replaced “testes” with exams.

The discussion is unnecessarily long. I recognize that many interventions were examined, and it is tempting to report and discuss all these interventions. However, the authors need to bring out the main message of the results and their implications for quality care.
Authors’ response:

We now submit a revised discussion focused on the main results. The interventions and screenings discussed are important tools for improving neonatal health in STP and similar countries. We did not discuss those related to maternal outcomes.

**Conclusion**
Provide a conclusion that is succinct and supported by the current findings

Authors’ response:

Done.

We now submit a new revised concise conclusion.

**Reviewer #2:**

The authors have done well to write the methods in detail but the study design and sampling method was not clearly explained. It would be good to give the total population size and proportion of female population to understand some of the statistics in a better way.

Authors’ response:

Done. New information was included in the Materials and Methods section.

In this revised paper we submit a new title as we believe that it better describes and addresses the global picture of this study:

Title: “Antenatal screenings and maternal diagnosis among pregnant women in Sao Tome & and Principe - missed opportunities to improve neonatal health: a hospital-based study”

Though it was a chart review study but the way the source documents were obtained is unclear.

Authors’ response:

Done. New information was included.

The tables need improvement. Authors’ response:

Done. We merged Tables 3, 4 and 5 into a revised new Table 1 for clearer comparison between data.

Statistical tests could also be done for group-wise comparison (e.g according to number of visits).

Authors’ response:

We appreciate the suggestion and included the comparison with p-values from the Chi-square test applied.

The subject may be locally relevant.

Authors’ response:

This paper will be very important for Sao Tome & Principe. We also believe that this study can be a model for other similar low-resource countries for the detection of the main gaps in the country´s ANC service enabling the design of target intervention and better allocation of resources to where they are needed.
