# Supplementary material for: Antenatal screenings and maternal diagnosis among pregnant women in Sao Tome & Principe—Missed opportunities to improve neonatal health: A hospital-based study
Source: PLOS Glob Public Health. 2022 Dec 29;2(12):e0001444. doi: 10.1371/journal.pgph.0001444 (PMC10021443; doi:10.1371/journal.pgph.0001444)
Supplement: Attachment — Submitted filename: Response to Reviewers PLOS_ANC_2nd round_22-00754.docx [file pgph.0001444.s003.docx]

Rebuttal letter

Point-by-point response to Reviewers

Dear Editor and Reviewers,

On behalf of all authors, we express our gratitude to you for the constructive review of our manuscript PGPH-D-22-00754R1

Antenatal screenings and maternal diagnosis among pregnant women in Sao Tome & Principe - missed opportunities to improve neonatal health: a hospital-based study

PLOS Global Public Health

We truly appreciate the Editor and Reviewer´s feedback for the first round and contributions fort his second round.

We have now revised the manuscript based on the comments raised by the Reviewer 2.

In the following detailed response, we address each comment calling for changes point-by-point, indicating where relevant additional texts have been added to the body of the manuscript.

We submit a manuscript with track changes and a clean version of the review manuscript.

We believe that with the changes made, the manuscript has improved substantively in view of being considered for publication.

___________________________________________________

Journal Requirements:

1. Please review your reference list to ensure that it is complete and correct. If you have cited papers that have been retracted, please include the rationale for doing so in the manuscript text, or remove these references and replace them with relevant current references. Any changes to the reference list should be mentioned in the rebuttal letter that accompanies your revised manuscript. If you need to cite a retracted article, indicate the article’s retracted status in the References list and also include a citation and full reference for the retraction notice.

Authors’ response:

Done.

2. Please amend your detailed Financial Disclosure statement. This is published with the article. It must therefore be completed in full sentences and contain the exact wording you wish to be published.

a. State the initials, alongside each funding source, of each author to receive each grant.

b. If any authors received a salary from any of your funders, please state which authors and which funders.

If you did not receive any funding for this study, please simply state: “The authors received no specific funding for this work.”

Authors’ response:

We amended the Financial Disclosure statement.

Funding

AV was funded by the Fundação para a Ciência e Tecnologia (FCT) (https://www.fct.pt/index.phtml.pt/), grant number SFRH/BD/117037/2016. The funder had no role in the study design, data collection and analysis, decision to publish or preparation of the manuscript.

Additional Editor Comments (if provided):

**Reviewers' comments:**

Reviewer's Responses to Questions

Comments to the Author

**6. Review Comments to the Author**

Please use the space provided to explain your answers to the questions above. You may also include additional comments for the author, including concerns about dual publication, research ethics, or publication ethics. (Please upload your review as an attachment if it exceeds 20,000 characters)

**Reviewer #1:**

Thank you for your time in addressing the comments raised. I deem the revised manuscript scientifically suitable for consideration for publication in PlOS Global Public Health

Authors’ response:

We really appreciate the Reviewers 1 precious contributions and feedback. We do believe that this paper will be very important for Sao Tome & Principe and similar countries.

**Reviewer #2:**

The authors have revised the manuscript to align with the comments from all reviewers. However, discussion is still quite lengthy. The authors are advised to limit discussing all the factors and retain only for the most important ones and keep one paragraph to discuss the rest. Of course, the authors will have to be mindful of the local context but a revision to shorten the text will be better.

Authors’ response:

We agree with the Reviewer 2 comment and we now submit a more concise discussion focused on the main results.

We tried to keep the interventions and screenings that are the most important tools for improving neonatal health in STP and similar countries.

The following cited papers that have been retracted, due to this revision:

55. DeBaun MR, Galadanci NA. Sickle cell disease in subSaharan Africa. 2019 Available at: https://www.uptodate.com/contents/sickle-cell-diseasein-sub-saharan-africa [Last accessed 2021 October 11].

56. Makani J, Moshi G. Haematology in sub-Saharan Africa: advances and opportunities in health care, education, and research. The Lancet Haematology. 2021;8(10):e678-81.

57. Esoh K, Wonkam-Tingang E, Wonkam A. Sickle cell disease in sub-Saharan Africa: transferable strategies for prevention and care. The Lancet Haematology. 2021
